# Disease Burdens Associated with PM_2.5_ Exposure: How a New Model Provided Global Estimates

**DOI:** 10.1289/ehp.122-A111

**Published:** 2014-04-01

**Authors:** Carrie Arnold

**Affiliations:** Carrie Arnold is a freelance science writer living in Virginia. Her work has appeared in *Scientific American*, *Discover*, *New Scientist*, *Smithsonian*, and more.

Like traffic jams and cell phones, particulate air pollution is a reality of modern living. Whether it’s from cigarette smoking, industrial emissions, or the burning of wood and dung for fuel, fine particulate matter (PM_2.5_) has been strongly linked to cardiovascular disease, inflammation, lung cancer, and other lung diseases.^1^^,^^2^ As part of the Global Burden of Disease Study (GBD) 2010 collaboration,^3^ an international team of environmental health scientists estimated the worldwide disease burden attributable to PM_2.5_ exposure.^4^ In this issue of *EHP*, they explain the underpinnings of how they did it.^5^

“This study is quite an important achievement,” says Robert Brook, a physician at the University of Michigan who was not involved with the research. “Currently, scientists have no other way to estimate these risks.”

**Figure:**
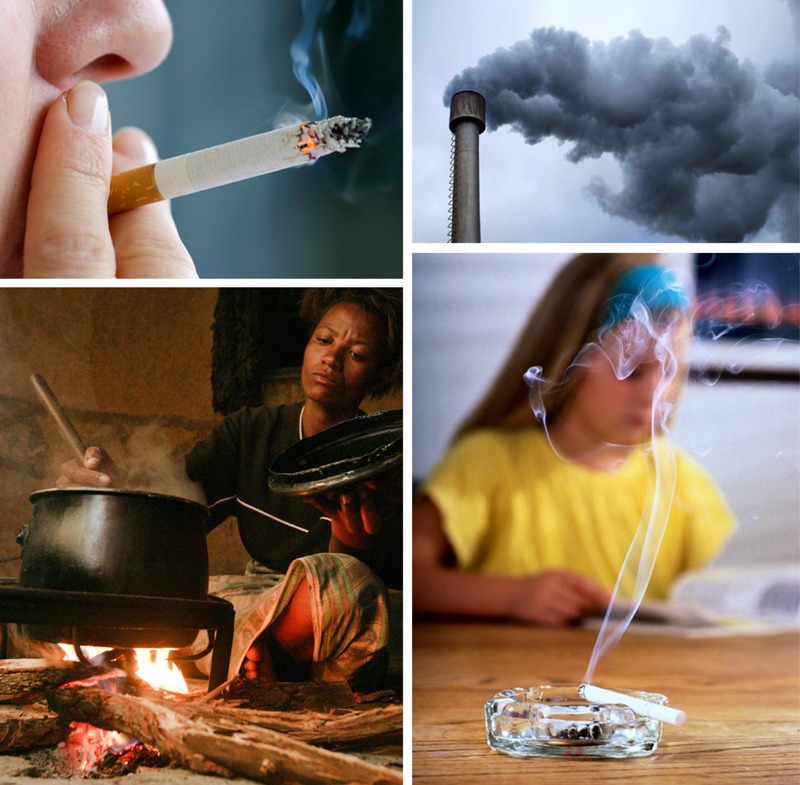
The GBD 2010 team incorporated information about the health risks of PM_2.5_ from ambient air pollution, active smoking, secondhand smoke, and indoor burning of solid fuels into an integrated exposure–response model. Clockwise from upper right: © Zirafek/iStockphoto; © David Young-Wolff/Getty; © Crispin Hughes/Panos; © Mac99/iStockphoto

In the past, risk models for PM_2.5_ and other air pollutants have been derived from work done almost exclusively in North America and Europe. But air pollution levels around the world vary widely—by one estimate, annual average PM_2.5_ concentrations in the United States top out at 20–40 µg/m^3^ (in Texas), compared with 80–130 µg/m^3^ in parts of China and India.^6^

Estimating the health risks of PM_2.5_ in developing countries, where few primary epidemiological studies have been conducted, has therefore required extrapolation to higher concentrations of air pollutants.^7^ Further complicating matters is evidence that the relationship between mortality risk and PM_2.5_ exposure is not linear, meaning risk appears to increase more rapidly at lower exposures than at higher ones.^8^^,^^9^

The GBD 2010 team incorporated information about the health risks of PM_2.5_ from ambient air pollution, active smoking, secondhand smoke, and indoor burning of solid fuels into an integrated exposure–response model. The amounts of pollution caused by smoking, secondhand smoke, and indoor fuel use are well documented, as are the resulting health problems.^10^^,^^8^^,^^11^

The researchers combined this knowledge with what is known about different concentrations of ambient air pollution around the world to estimate relative risks of dying from a variety of illnesses, including ischemic heart disease, cerebrovascular disease (stroke), chronic obstructive pulmonary disease, and lung cancer over a range of ambient PM_2.5_ exposures from very low to very high. The model also estimates years of healthy life lost due to PM_2.5_-related acute lower respiratory illnesses in children under the age of 5 years.^5^

“We integrated as much information on exposure to particulate matter and mortality from as many different sources as we possibly could,” says lead author Richard Burnett, an environmental scientist at Health Canada. “Because some of these exposures had very high concentrations, we could build a risk model over the entire global range of potential exposures.”

The risk curves for different illnesses were not identical. Whereas the estimated risk of lung cancer and child acute lower respiratory illness appeared to continually increase along with increasing concentrations of PM_2.5_, the curves for heart disease and stroke rapidly increased at lower concentrations of PM_2.5_ before leveling off.^5^

One of the major advantages of the model is that it can be updated easily, yielding estimates of risk that reflect the latest knowledge. The major limitation of the study is that the researchers assumed the health risks of different types of PM_2.5_ were the same, says Michael Jerrett, a professor of environmental health sciences at the University of California, Berkeley, who was not involved with the study.

PM_2.5_ is a mixture of many constituents that can vary, depending on the source and the season.^12^ “There is considerable evidence that some elements of the mixture are less toxic than others,” Jerrett says. “However, to try to untangle that and come up with definitive estimates for sulfate versus nitrate versus black carbon would go beyond the capacity of our current scientific evidence to determine accurately.” He concludes, “It’s a mathematically elegant model that is a major advance over what has been done in the past. It represents the best that we can do with the available information.”
